# Efficacy and Safety of Different Doses of Bevacizumab Combined With Pemetrexed and Platinum in First-Line Treatment of Advanced NSCLC: A Retrospective-Real World Study

**DOI:** 10.3389/fphar.2021.727102

**Published:** 2021-11-17

**Authors:** Chun-Hua Zhou, Feng Yang, Wen-Juan Jiang, Yong-Chang Zhang, Hai-Yan Yang, Liang Zeng, Li Liu, Yi Xiong, Fan-Xu Zeng, Zhan Wang, Nong Yang

**Affiliations:** ^1^ Lung Cancer and Gastrointestinal Unit, Department of Medical Oncology, Hunan Cancer Hospital/The Affiliated Cancer Hospital of Xiangya School of Medicine, Central South University, Changsha, China; ^2^ Center of New Drug Clinical Trial, Hunan Cancer Hospital and The Affiliated Cancer Hospital of Xiangya School of Medicine, Central South University, Changsha, China

**Keywords:** bevacizumab, chemotherapy, different dose, efficacy and safety, NSCLC

## Abstract

**Background:** Bevacizumab was demonstrated to have efficacy in patients with NSCLC. However, application of different doses of bevacizumab in different clinical trials was overlooked. This study aims to investigate the effects and safety of different doses of bevacizumab in the treatment.

**Methods:** From January 2016 to March 2020, 79 patients with NSCLC received first-line combination treatment with chemotherapy (pemetrexed + platinum) and bevacizumab for four cycles; patients without progression after four cycles were randomly assigned to maintenance therapy with bevacizumab combined with pemetrexed, of which 57 patients received bevacizumab at a dose of 7.5 mg/kg and 22 patients at a dose of 15 mg/kg. The primary endpoint was progression-free survival, and secondary endpoints were overall response rate, disease control rate, and adverse events.

**Results:** There was no significant difference between two groups in effectiveness; Median PFS in 7.5 mg/kg group and in 15 mg/kg group were 8.0 and 8.7 months, respectively (*p* = 0.663), reaching the primary endpoint. The ORR and DCR in the bevacizumab 7.5 and 15 mg/kg group were 45.46 and 86.0% vs. 50 and 90.9% showing no statistical significance (*p* = 0.804 and 0.717). Most of side effects were tolerable. The incidences of overall toxicities were higher in 15 mg/kg group (*p* = 0.001). No new safety signals were observed.

**Conclusion:** We did not detect significant difference of efficacy and safety between 7.5 mg/kg group and 15 mg/kg group for bevacizumab administration, the cost-effectiveness of the 7.5 mg/kg group was significantly better than that of the 15 mg/kg group.

## Introduction

Lung cancer is the most common cancer with leading cause of cancer deaths in China and worldwide (Chen et al., 2016; Siegel et al., 2018, 2019). In recent years, with the emerging of the tyrosine kinase inhibitors (TKIs) for patients with EGFR mutation, ALK rearrangement or ROS1 rearrangement, and immune checkpoint inhibitors, the treatment for advanced NSCLC has changed greatly and the 5-years survival rate of patients has also been greatly improved. However, the use of targeted therapy may be restricted to patients whose tumor has a specific gene mutation and the application of immune monotherapy requires specific immune markers, and the ORR is limited (Reck et al., 2019). In recent years, with the effectiveness of anti-angiogenesis therapy, the prognosis of patients with NSCLC has been greatly improved. Bevacizumab is a humanized immunoglobulin G monoclonal antibody blocking VEGF-mediated signaling pathways and thus angiogenesis, so as to play an effective role of anti-tumor (Hanna et al., 2020).

Several clinical trials have confirmed that the combination of bevacizumab and chemotherapy (BC) significantly prolonged progression free survival (PFS) and overall survival (OS) in NSCLC patients as compared with chemo-monotherapy (Crinò et al., 2010; Zhou et al., 2015; Rocco et al., 2019). BC has become the standard first-line clinical treatment for non-squamous NSCLC without driver gene mutations. However, the dose of bevacizumab was inconsistent in different studies. In ECOG4599, BEYOND and Pronounce, the dose of bevacizumab was used by 15 mg/kg, while 7.5 mg/kg was used in MO22089, and they were both with good overall efficacy and safety (Barlesi et al., 2013; Zhou et al., 2015; Zinner et al., 2015; Li et al., 2019).

There are few comparative studies of bevacizumab in NSCLC. Most treatment guidelines did not make a recommendation of the optimal dose to physicians. In AVAIL, bevacizumab was used by 7.5 or 15 mg/kg in BC regimen. The treatment efficacy of both bevacizumab groups seemed to be similar when compared to the placebo group. However, further analysis of 105 Asian patients in the study showed that there was OS benefit in the low-dose bevacizumab group, but not in the high-dose bevacizumab group. In terms of safety, the incidence of grade 3 or above adverse reactions in high-dose bevacizumab group was 44%, which was higher than that in low-dose bevacizumab group (33%) and chemotherapy group (33%) (Reck et al., 2009). Therefore, for Asian patients, low-dose bevacizumab combination therapy may be able to obtain better efficacy.

In this study, all patients received first-line bevacizumab plus pemetrexed-platinum (PC). In the JMDB study, PC was the preferred chemotherapy regimen for non-small cell lung cancer (Scagliotti et al., 2008). Moreover, many studies demonstrated that the combination of bevacizumab with PC was well tolerated in the treatment of NSCLC (Barlesi et al., 2013; Patel et al., 2013; Zinner et al., 2015; Xu et al., 2016; Shan et al., 2018; Tsutani et al., 2018; Kreis et al., 2019). We conducted the assessment of treatment with real world study to compare the efficacy and safety of low (7.5 mg/kg) and high (15 mg/kg) dose of bevacizumab in combination with PC in advanced non-squamous NSCLC.

## Patients and Methods

### Patients

A total of 1,205 treatment-naïve patients were diagnosed with advanced NSCLC from January 2016 to March 2020 at Hunan Cancer Hospital. After screening, all patients were ≥18 years old and histologically diagnosed of non-squamous NSCLC with stage IV. They were all with an Eastern Cooperative Oncology Group (ECOG) performance status (PS) of 0–1. Their driver gene mutations were negative and first line treatments were BC. The characteristics of the patients including sex, age, smoking history, brain metastasis, and status of HER-2, BRAF, KRAS, TP53, M861 mutations are summarized in Table 1.

**TABLE 1:** Characteristics of NSCLC patients.

Variables	Bev 7.5 mg/kg (%)	Bev 15 mg/kg (%)	χ^2^	*P*
Total NO. of patients	57	22	—	—
Age			1.042	0.328
Median	59.8	59.1	—	—
Range	33–76	45–69	—	—
≥60	29 (50.9)	8 (36.4)	—	—
<60	28 (49.1)	14 (63.6)	—	—
Sex			0.239	0.780
Male	42 (73.7)	15 (68.2)	—	—
Female	15 (26.3)	7 (31.8)	—	—
Smoking status			0.883	0.425
Yes	40 (70.2)	13 (59.1)	—	—
No	17 (29.8)	9 (40.9)	—	—
Brain metastasis			0.007	0.860
Yes	7 (12.3)	3 (13.6)	—	—
No	50 (87.7)	19 (84.4)	—	—
Gene mutation^a^			0.026	0.161^b^
Yes	15 (26.3)	6 (27.3)	—	—
No	42 (73.7)	16 (72.7)	—	—

a Gene mutations: HER-2, BRAF, KRAS, TP53, M861 mutations.

b One data grid is expected to be less than 5, which is calculated by the standardized method.

### Treatment

Patients received induction therapy on day 1 of each 21-day period by the regimen of cisplatin (75 mg/m2)/carboplatin (area under the curve, AUC 6), pemetrexed (500 mg/m2), and bevacizumab. Induction therapy was repeated every 3 weeks for a maximum of four cycles. After completion of at least three cycles of induction therapy, patients received maintenance therapy on day 1 of the 21-days cycle comprising pemetrexed (500 mg/m2) and bevacizumab. The dose of bevacizumab in maintenance therapy was the same as induction therapy. Maintenance therapy was repeated every 3 weeks until disease progression or intolerance.

### Assessment

Chemotherapy response was evaluated after every two treatment cycles by computed tomography. They were evaluated as complete response (CR), partial response (PR), stable disease (SD), progression disease (PD), or not evaluable according to the Response Evaluation Criteria in Solid Tumor criteria 1.1.9. The ORR was defined as the sum of CR and PR. The disease control rate (DCR) was defined as the sum of CR, PR, and SD. Toxicities were evaluated according to the National Cancer Institute Common Terminology Criteria for Adverse Events version 5.0. The primary endpoint was progression-free survival (PFS). Secondary endpoints were ORR, DCR, and adverse effect.

### Statistics Analysis

Descriptive summaries were created for demographic and clinical variables. The Chi-squared test was used to compare subset variables and toxicities. All *p* values were two-tailed. Kaplan–Meier curves were generated for progression free survival and overall survival. Log-rank tests were used to compare the survival between groups. All statistical analyses were performed using the SPSS 22.0 software for Windows (SPSS Corp., Armonk, NY, United States); *p* < 0.05 was considered to indicate a statistically significant difference.

## Results

### Patient Characteristics

**FIGURE 1:**
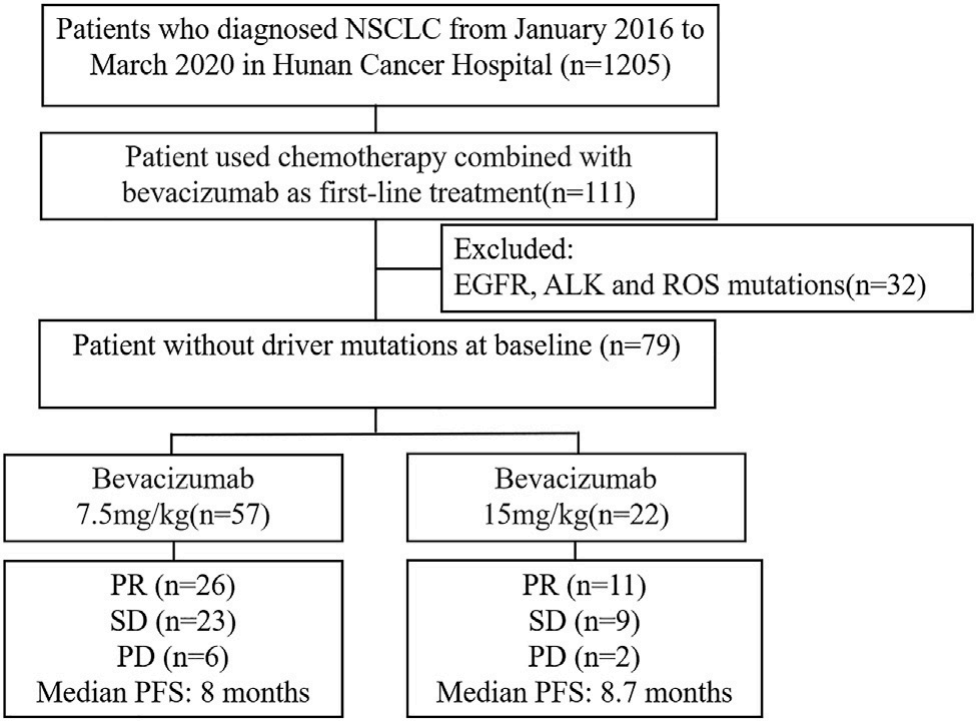
Flow diagram of sample screening.

Among 1,205 NSCLC patients, 111 patients who received first-line PC combined with bevacizumab were enrolled in this study. Thirty-two patients with EGFR mutations, ALK fusions, or ROS-1 fusions were excluded (Figure 1). A retrospective analysis was performed in 79 NSCLC patients who had received first-line treatment of bevacizumab combined with PC, and 57 of them received bevacizumab at a dose of 7.5 mg/kg and 22 patients received bevacizumab at a dose of 15 mg/kg. The characteristics of the patients, including sex, age, smoking history, brain metastasis, and status of gene mutations, are summarized in Table 1. There were no significant differences in the baseline characteristics. According to the TNM classification for NSCLC patients (AJCC 7th), all patients were diagnosed with stage IV lung adenocarcinoma.

### Clinical Efficacy

The treatment responses are listed in Table 2. There was no patient who achieved CR in the whole population. Of the 57 patients in treatment with bevacizumab 7.5 mg/kg group, 26 (45.6%) achieved PR, 23 (40.4%) achieved SD, and 6 (10.5%) achieved PD; and of the 22 patients in the treatment with bevacizumab 15 mg/kg group, 11 (50.0%) achieved PR, 9 (40.9%) achieved SD, and 2 (9.1%) achieved PD. The ORR and DCR in the bevacizumab 7.5 mg/kg group were 45.46 and 86.0% while in bevacizumab 15 mg/kg group were 50 and 90.9%, respectively. There is no significant difference in the treatment efficacy between patients with different doses of bevacizumab (ORR, *p* = 0.804; DCR, *p* = 0.717).

**TABLE 2:** Summary of treatment response.

Response	7.5 mg/kg group (*n* = 57) (%)	15 mg/kg group (*n* = 22) (%)	*P* value
CR	—	—	—
PR	26 (45.6)	11 (50.0)	0.348
SD	23 (40.4)	9 (40.9)	0.964
PD	6 (10.5)	2 (9.1)	0.188
NE	2 (3.5)	—	—
ORR	26 (45.6)	11 (50.0)	0.348
DCR	49 (86.0)	20 (90.9)	0.589

CR, complete response; DCR, disease control rate; NE, not evaluable; ORR, overall response rate; PD, progressive disease; PR, partial response; SD, stable disease.

**FIGURE 2:**
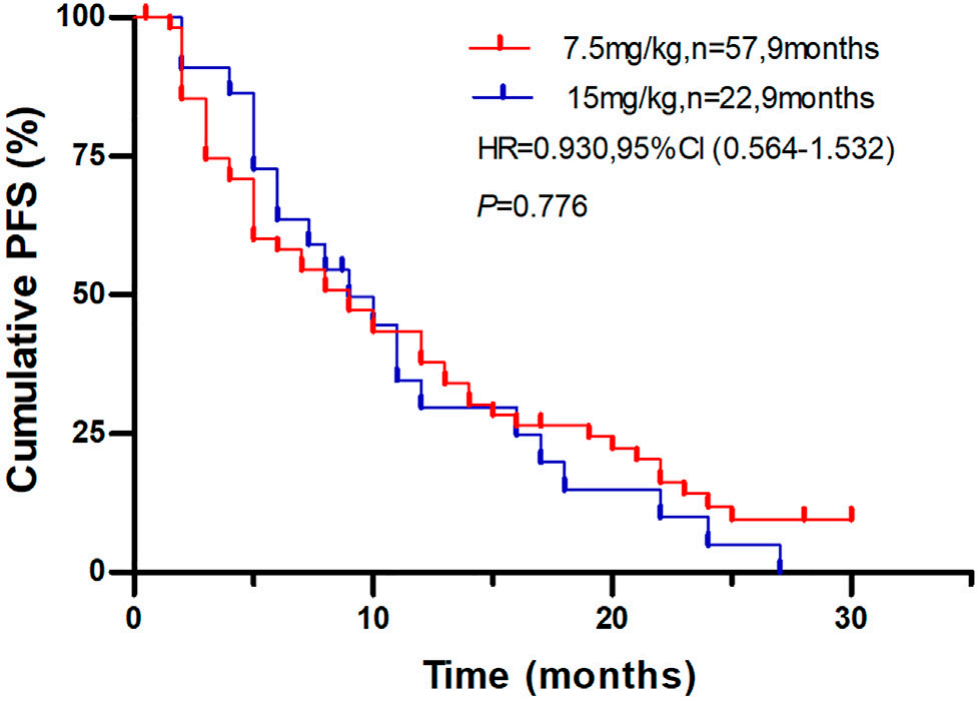
Plot of Kaplan-Meier estimates of progression-free survival for the bevacizumab 7.5 mg/kg group compared with 15 mg/kg group.

**FIGURE 3:**
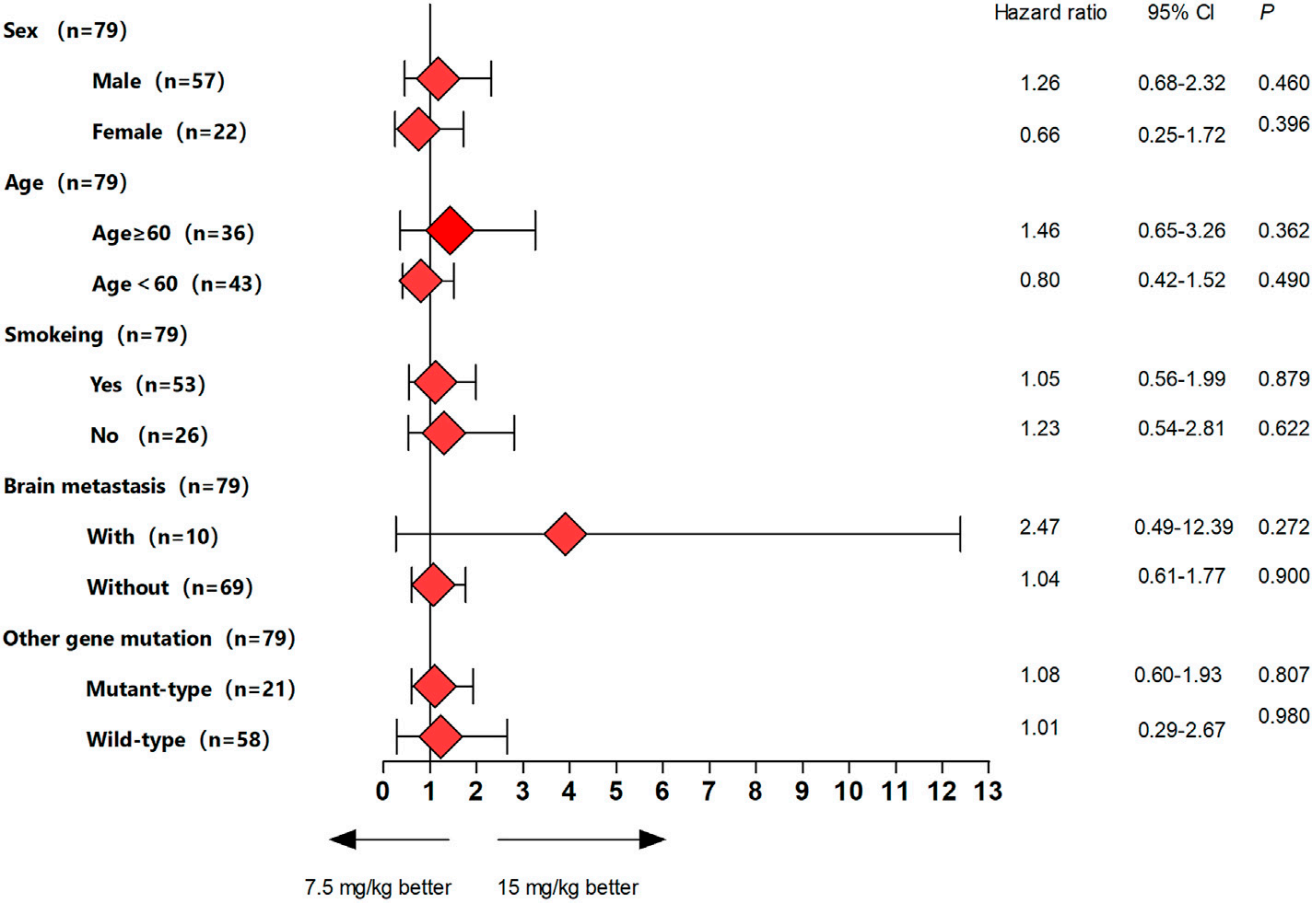
Forest plots of hazard ratios for progression-free survival by subgroups for each bevacizumab group.

This study met the primary endpoint. The median PFS (mPFS) were 8.0 months (95% CI, 4.8–11.1 months) in the bevacizumab 7.5 mg/kg group and 8.7 months (95% CI, 5.5–11.8 months) in the bevacizumab 15 mg/kg group, respectively (Figure 2). There was no significant difference between the two groups (*p* = 0.766). Moreover, we conducted subgroup analysis according to the clinical variables, and the results showed no significant differences between bevacizumab 7.5 mg/kg group and bevacizumab 15 mg/kg groups (Figure 3).

### Toxicity

Most side effects were tolerable (grade 1–2); nine patients (11.4%) had severe adverse events (grade 3–4). As summarized in Table 3, the most common adverse events were leukopenia (26.5%) and liver dysfunction (22.8%) in bevacizumab 7.5 mg/kg group, while leukopenia (40.9%) and proteinuria (22.7%) in bevacizumab 15 mg/kg group. For the grade 3–4 adverse events, the frequencies of hypertension, leukopenia, thrombocytopenia, vomiting, and liver dysfunction were 3.5, 1.8, 1.8, 1.8, and 1.8% in bevacizumab 7.5 mg/kg group, respectively. The frequencies of grade 3–4 adverse events, including leukopenia, nausea, vomiting, thrombocytopenia, fatigue, and proteinuria, were 9.1, 9.1, 9.1, 4.5, 4.5, and 4.5% in bevacizumab 15 mg/kg group, respectively. The incidence of overall toxicities in 15 mg/kg group is statistically higher than 7.5 mg/kg group (*p* = 0.001) (Table 4). Meanwhile, the subgroup comparisons of G3-4 adverse events, the rate of leukopenia, and liver dysfunction had been done. However, there was no statistically significant difference of these adverse between two groups. No new safety signals were observed in this study.

**TABLE 3:** Treatment related adverse events.

Adverse events	Bev 7.5 mg/kg (n = 57)			Bev 15 mg/kg (n = 22)		
	Grade 1–2 n (%)	Grade 3–4 n (%)	Total n (%)	Grade 1–2 n (%)	Grade 3–4 n (%)	Total n (%)
Leukopenia	14 (24.6)	1 (1.8)	15 (26.3)	7 (31.8)	2 (9.1)	9 (40.9)
Anemia	1 (1.8)	0	1 (1.8)	1 (4.5)	0	1 (4.5)
Thrombocytopenia	1 (1.8)	1 (1.8)	2 (3.5)	0	1 (4.5)	1 (4.5)
Nausea	1 (1.8)	0	1 (1.8)	0	2 (9.1)	2 (9.1)
Fatigue	0	0	0	0	1 (4.5)	1 (4.5)
Vomiting	2 (3.5)	1 (1.8)	3 (5.3)	0	2 (9.1)	2 (9.1)
Liver dysfunction	12 (21.0)	1 (1.8)	13 (22.8)	3 (13.6)	0	3 (13.6)
Hemoptysis	2 (3.5)	0	2 (3.5)	0	0	0
Hypertension	0	2 (3.5)	2 (3.5)	1 (4.5)	0	1 (4.5)
Proteinuria	4 (7.0)	0	4 (7.0)	4 (18.2)	1 (4.5)	5 (22.7)
Abdominal pain	1 (1.8)	0	1 (1.8)	0	0	0
Renal dysfunction	3 (5.3)	0	3 (5.3)	0	0	0
Edema	1 (1.8)	0	1 (1.8)	0	0	0

**TABLE 4:** Comparison of adverse events.

Adverse events	Bev 7.5 mg/kg	Bev 15 mg/kg	*P* value
	N (%)	N (%)	
Total	21 (36.8)	17 (77.3)	0.001
Grade 3–4	4 (7.0)	5 (22.7)	0.106^a^
Leukopenia	15 (26.3)	9 (40.9)	0.465^a^
Liver dysfunction	13 (22.8)	3 (13.6)	0.751^a^
Proteinuria	4 (7.0)	5 (22.7)	0.106^a^

a Fisher’s Exact Test was applied.

## Discussion

Bevacizumab has been widely used in anti-tumor therapy in the form of monotherapy and combination therapy. However, there is no standard dosage for bevacizumab. Fatih Kose et al. reported the half-dose bevacizumab experience in relapsed ovarian cancer patients, following showed the lower dose group has similar effectiveness with lower rate of hypertension (Kose et al., 2020). In NSCLC, serval clinical trials were designed with the selected bevacizumab dose arms or proceeding subgroup analysis. There were few bevacizumab dose comparative studies that have been reported.

The immune checkpoint inhibitors (ICIs) have dramatically changed the landscape of NSCLC treatment. Bevacizumab has been investigated in combination with immunotherapy and chemotherapy. Based on IMPOWER 150 study, combination therapy with atezolizumab, bevacizumab, and chemo [carboplatin + paclitaxel (CP)] has been approved for first line treatment in advanced non-squamous NSCLC. In exploratory analysis of IMPOWER 150, the arms containing bevacizumab (ABCP and BCP) had lower rate of new brain lesions comparing with ACP arm ACP (ACP 11.9%, *n* = 48, ABCP 7%, *n* = 28, BCP 6%, *n* = 24) (Socinski et al., 2018). On the other hand, the dosage of bevacizumab in this trail (15 mg/kg) should be noticed, because the incidence of AEs leading to bevacizumab withdrawal (13%) was higher than chemo (6%) and atezolizumab (8%) (Dhillon and Syed, 2019). However, we do not have enough immuno-combination therapy cases to proceed with comparative research. In this real-world study, we compared the efficacy and safety of bevacizumab used by 7.5 or 15 mg/kg in stage IV non-squamous NSCLC patients, who were treated by cisplatin/carboplatin, pemetrexed, and bevacizumab. It would offer a reference to improve the effectiveness and safety of immuno-combination therapy which contains bevacizumab.

The results of this study revealed that there was barely noticeable difference of mPFS between 7.5 mg/kg group and 15 mg/kg group. The ORR and DCR were 45.46 and 86.0% in bevacizumab 7.5 mg/kg group and were 50 and 90.9% in bevacizumab 15 mg/kg group, which was consistent with historical research data (Zhou et al., 2014). In AVAPERL (MO22089), patients with advanced NSCLC received first-line bevacizumab 7.5 mg/kg, the mPFS was 7.4 months (Barlesi et al., 2013); while in e COMPASS (WJOG5610L) and Point Break trial patients received bevacizumab 15 mg/kg, the mPFS was 6.0 months (Zinner et al., 2015; Kreis et al., 2019). AVAIL study shares some similar conclusions with our study. The study compared the treatment results of 7.5 or 15 mg/kg of bevacizumab versus placebo in combination with chemotherapy, and confirmed the superiority of bevacizumab maintenance treatment over placebo. The median PFS of bevacizumab 7.5 mg/kg group and bevacizumab 15 mg/kg group were 6.7 vs. 6.5 months, which was consistent with our data. However, the direct comparison between 7.5 and 15 mg/kg was not performed. In addition, although AVAIL study shows that the overall incidence of serious AEs was higher in the 15 mg/kg bevacizumab plus CG arm (44%) compared with the placebo plus CG and 7.5 mg/kg bevacizumab plus CG arms, the chemotherapy regimens administrated in AVAIL study is gemcitabine, while the current superior regimen for the treatment of lung adenocarcinoma is pemetrexed (Huang et al., 2012; Shi et al., 2013; Hu et al., 2016). It is worth noting that most of historical clinical trials mentioned above did not compare the efficacy and safety of the two doses of bevacizumab directly. In our study, the overall incidence of AEs was higher in the high-dose group. Since the majority of AEs reported during this study were leukopenia, liver dysfunction, and proteinuria, we compared the rate of these selected AEs in two groups. The incidence of liver dysfunction was higher in the7.5 mg/kg group and may result from the small sample size of 15 mg/kg group. Moreover, the incidence of proteinuria which mainly arising from bevacizumab was much higher in the 15 mg/kg group.

The baseline characteristics are mostly identical in both groups, but for the age of ≥60 years old, the percentage in high dose group was much lower, and we did propensity score matching (PSM 1:2 match on the nearest neighbor) to balance the covariate. Matched sets of low-dose group patients (*n* = 44) and high dose group patients (*n* = 22) who share a similar value of the propensity score of age was analyzed. We did not find any significant difference (*p* = 0.864); the result is consistent with that in overall sample.

There are some limitations in this study. Firstly, the sample size is small and may increases the likelihood of a Type II error, which decreases the power of the study. Secondly, several patients crossed over from high dose group to low dose group in maintenance therapy, and that may affect the results of side-effects analysis. It is necessary to research whether the dose of maintenance therapy is consistent with that of induction therapy. Lastly, we conducted subgroup analyses of PFS in the two groups according to the clinical variables, but no significant differences were observed in all the subgroups. It might be because the sample size was too small, and a larger sample size is needed in further investigations.

In conclusion, chemotherapy combined with bevacizumab 7.5 and 15 mg/kg reach the similar PFS, ORR, and DCR in advanced non-driver gene mutation NSCLC patients. No significant difference of efficacy was observed between the two groups. However, the incidences of overall toxicities were higher in the 15 mg/kg group. Meanwhile, the medical fees of half-dose bevacizumab administration were significantly lower than that of 15 mg/kg; as a result, considering the cost-effectiveness, the 7.5 mg/kg administration was logically preferred.

## Data Availability

The original contributions presented in the study are included in the article/Supplementary Material, and further inquiries can be directed to the corresponding authors.
